# Patient and Provider Experience With Cystic Fibrosis Telemedicine Clinic

**DOI:** 10.3389/fped.2021.784692

**Published:** 2021-11-24

**Authors:** Kalen Hendra, Fatima Neemuchwala, Marilynn Chan, Ngoc P. Ly, Elizabeth R. Gibb

**Affiliations:** ^1^UCSF Benioff Children's Hospital Oakland, Oakland, CA, United States; ^2^Division of Pediatric Pulmonology, Department of Pediatrics, University of California, San Francisco, San Francisco, CA, United States

**Keywords:** cystic fibrosis, quality improvement, telehealth, telemedicine, patient experience, provider experience

## Abstract

In response to the novel coronavirus (COVID-19) pandemic, all in-person cystic fibrosis (CF) appointments were converted to telemedicine visits at UCSF Benioff Children's Hospital. The purpose of our study was to learn about the experiences that patients, families, and providers had with telemedicine visits and to assess their interest in using telemedicine in the future. Our hypothesis was that most patients, families, and providers want to continue telemedicine visits in the future. An anonymous 11-question survey was distributed to patients, families, and providers in November and December 2020. The survey was completed by 46 of 72 families (64% response rate) and 24 of 25 providers (96% response rate). Thirty-seven families (80%) and 21 providers (88%) were satisfied with their telemedicine experience. Thirty-three families (72%) want to have telemedicine visits in the future. Thirty-five families (76%) and 22 providers (92%) were satisfied with their experience using Zoom. Forty families (87%) and 19 providers (90%) want 2 or more visits each year to be via telemedicine. Our study showed that most families and providers were satisfied with telemedicine, would like to continue using telemedicine, and prefer to have at least 2 of the 4 recommended annual CF visits via telemedicine. Our survey identified the following benefits to telemedicine: decreased travel time, decreased cost, and avoiding exposure to COVID. However, we need to ensure that we do not exacerbate existing health disparities for families that do not speak English and/or do not have the internet capabilities to support telemedicine technology.

## Introduction

On March 11, 2020 the novel coronavirus disease (COVID-19) outbreak was declared a pandemic by the World Health Organization. Two days later, the United States declared COVID-19 a national emergency. In response, UCSF Benioff Children's Hospital Oakland and San Francisco converted all in-person Cystic Fibrosis (CF) appointments to telemedicine visits via Zoom, a secure web-based platform that provides video and telephone services. The first telemedicine CF visit was conducted on March 27, 2020.

Prior to the COVID-19 pandemic, telemedicine was generally thought of as a way to increase access to subspecialty care in rural and/or underserved areas. A prior study by Kane and Gillis found that only 15.4% of physicians worked in a practice that utilized telemedicine to interact with patients (1). In pediatrics, the percentage was even lower: only 11.8% of pediatric physicians worked in a practice that utilized telemedicine to provide patient care. The survey utilized in this study defined telemedicine as “the use of technology as a substitute for an in-person encounter with a health care professional.” Although the Kane and Gillis study found that video conferencing was the most commonly used telemedicine modality, there are many types of telemedicine including: telephone calls, facilitated virtual visits with examination equipment, remote monitoring of chronic disease via various measurements (e.g. glucose, blood pressure, weight, etc.), storage and forwarding of health data (e.g. imaging, photos of rashes), and patient portals (e.g. MyChart) (2).

In a systematic review to assess the utility of telemedicine to monitor symptoms, monitor adherence to prescribed therapies, and provide therapeutic intervention to adults and children with CF, the investigators found that monitoring symptoms and collecting objective data (e.g. spirometry data) via telemedicine is feasible in patients with CF. However, there was a high rate of non-compliance with data reporting; therefore, the benefits of telemedicine in CF patients could not be determined (3). Since that 2012 systematic review, the use of telemedicine in the care of patients with CF has changed dramatically, in large part due to the COVID-19 pandemic.

The COVID-19 pandemic forced us to utilize telemedicine to provide multidisciplinary medical care for many patients, including CF patients. Our study was designed to help us understand the telemedicine experience of patients with CF as well as CF care team members, and to assess their interest in continuing to use telemedicine in the future. Our hypothesis was that most patients, families, and providers want to continue telemedicine visits in the future.

## Materials and Methods

This cross-sectional study was performed at UCSF Pediatric Cystic Fibrosis Center. The study population consisted of CF patients (ages 0–22 years) and their families as well as CF care providers (including pulmonologists, gastroenterologists, pharmacists, social workers, respiratory therapists, physical therapists, dieticians, nurses, and pediatric residents).

Patients were seen in the multidisciplinary Cystic Fibrosis clinic virtually, using the Zoom platform with breakout rooms. Patients were scheduled with all providers on the same day. Interpreters were provided to all families when indicated. In-person visits with the CF care team were completed in limited cases as clinically indicated (e.g. sick patients). Patients were scheduled for separate in-person visits in the pulmonary function laboratory for pulmonary function tests (PFTs) and throat/sputum cultures when indicated.

Study participants were asked to complete either a patient or provider survey about their experience with telemedicine visits, experience using Zoom, interest in using telemedicine in the future, benefits of telemedicine, benefits of in-person visits, and the frequency in which they would like various measurements/diagnostic studies (e.g. height and weight, PFTs, throat cultures, vital signs, physical exams, labs, chest x-rays) to be obtained. The families of CF patients either received a link to the Qualtrics survey in English via email or had the survey administered verbally, either via Zoom or over the phone. Non-English-speaking families had the survey administered verbally with the assistance of a medical interpreter (*n* = 12). Families were sent reminders to fill out the survey via email and via text message. The 72 families who receive regular CF care at UCSF received the patient survey. A total of 25 CF providers received a link to the Qualtrics provider survey via email. All survey data were de-identified and collected from November to December 2020. Telemedicine visits were ongoing at the time the survey was conducted. At the time of the survey, patients had had more than one telemedicine visit and at least one visit within 3 months of the survey. The patient and provider surveys can be found in Supplemental Materials Appendices 1 and 2.

Per UCSF IRB, this study was exempt from further review.

## Results

### Clinic Metrics

Three hundred and sixty-two (362) encounters were entered into the Cystic Fibrosis Registry in 2019 and 375 encounters were entered in 2020. In 2019, the average length of in-person visits was 134 minutes. In 2020, telemedicine visits lasted between 30 and 90 minutes. Of our 72 CF patients followed in our center, only 4 patients (6%) were seen in-person and only 4 patients (6%) had audio-only telemedicine visits (due to difficulties with internet connection or trouble setting up Zoom) between March 2020 and January 2021. The percent of patients with at least 4 visits per year increased from 88% in 2019 to 95% in 2020. The only patients who did not have at least 4 visits were patients who moved in/out of our center during 2020 or were diagnosed with CF mid-year. All patients were seen by a respiratory therapist, dietitian, and social worker at least once in 2020. Ninety-seven percent of patients 7 years and older had at least one PFT and one culture. Ninety-six percent of our patients completed annual labs. The clinic no-show rate improved by 40% during the transition to telemedicine.

### Patient Survey

Of the 72 families who received the patient survey, 46 families responded (64% response rate). Of the 46 respondents, there were families with children age 0–24 months (*n* = 4), 2–5 years (*n* = 13), 6–12 years (*n* = 12), 13–17 years (*n* = 10), and older than 18 years (*n* = 7).

Of the 46 respondents, 37 (80%) were overall satisfied with their telemedicine visits. Forty-one respondents (91%) felt that all their questions and concerns were addressed during their telemedicine visits. Thirty-five respondents (76%) were satisfied with their experience using Zoom. However, seven reported having internet connection difficulties and two reported having difficulty using or locating the Zoom ID.

**Figure 1:**
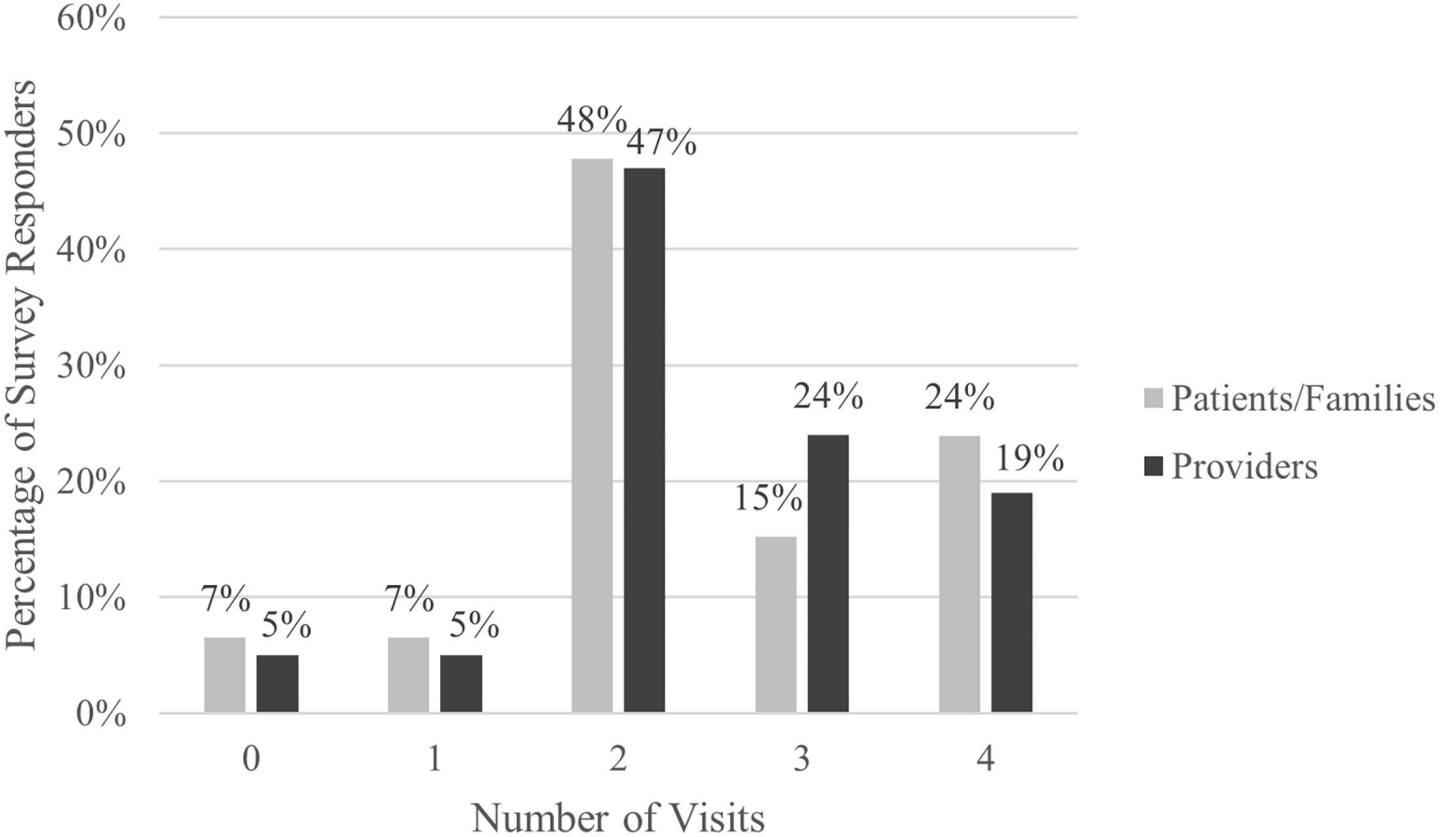
Number of telemedicine visits desired per year.

Thirty-three respondents (72%) indicated that they would like to have future visits via telemedicine. When specifically asked about the number of telemedicine visits they preferred per year, 47% of families preferred 2 out of 4 recommended annual CF visits be provided virtually. Forty families (87%) wanted 2 or more visits each year to be telemedicine visits (Figure 1). However, there were 3 families (7%) who reported that they wanted all 4 visits to be in-person.

Respondents identified the following benefits of telemedicine visits: decreased cost, decreased travel time, decrease in the amount of time missing work/school, do not have to arrange for childcare for other children, concerns about COVID, and concerns about poor air quality from fire smoke. Decreased travel time and concerns about COVID were the two most common reasons that families preferred telemedicine visits. One respondent commented that they “felt the doctors were more focused,” “there was less waiting,” and the “kids did not get bored.”

The most common reasons for wanting an in-person visit were: to have a physical exam done and to complete PFTs, labs, throat culture, etc. on the same day. Some felt an in-person visit was more personal (*n* = 12). Two respondents reported that they preferred in-person visits because they experienced technical difficulties. Families also preferred in-person visits if their child was sick or if there was an urgent matter. Additional comments from the patient surveys can be seen in Table 1.

**Table 1:** Comments and suggestions from the patient survey.

*Reasons families would like to have telemedicine visits*
If I don't need to bring the kids in because they are not sick, I can have the appointment via telemedicine.
I have concerns about exposure. I also have concerns that through televisions the care is not acceptable.
Feel the doctors are more focused – seems to be less waiting around and kiddos don't get bored!
Driving to San Francisco makes me nervous.
*Reasons families would like to have in-person visits*
I feel that the staff get to know our son better and build a stronger relationship over time.
If my child is feeling sick.
*Additional comments*
I prefer when the doctors are together in the appointment so it is shorter. Otherwise I do like my child to see everyone.
Having everything possible sent to PCP for PCP to do like throat swabs.
I think weight and height are important but it is easy to do at home. Overall I've had a great experience with telemedicine visits.
A lot of the information can be given ahead of time – would maybe be helpful to know when the nurse is going to call pre-meeting for info or send a secure form for us to fill in with height, weight, any issues, etc.
I'm happy with these visits. They are thorough and convenient.
I would like to thank the doctors for taking care of my kids.
I would like all visits to be in person.
I would like to alternate video visits with in-person visits. I feel more secure with a physical exam that my daughter is OK.
If there is an urgent matter I would like to have an in-person appointment. If it is not urgent, I am ok with a telephone visit.
We would like to return to in-person visits whenever possible.
Given CF patients are at high risk and you cannot run clinics at full capacity, it would be great to have at least 2 of the 4 annual visits in person. It makes me nervous as a parent to have my child not seen in person because this disease can creep up on you and I really want to make sure we are staying ahead of it, especially given the coronavirus, and knowing it's not going away anytime soon.
Everything goes well.
Parking is too expensive.

**Figure 2:**
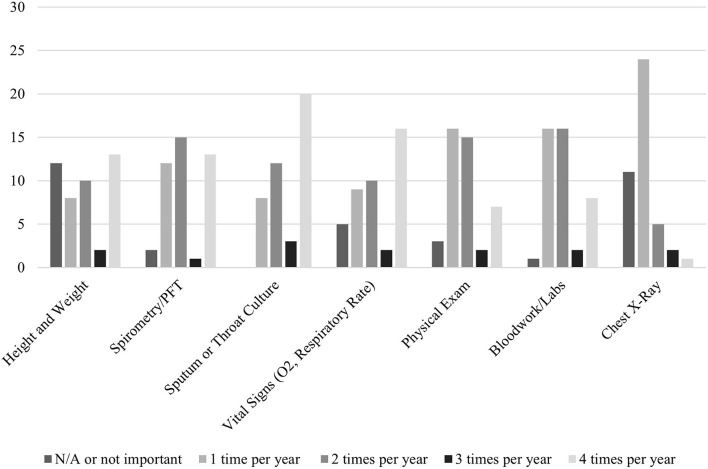
Frequency of obtaining clinical data as desired by families.

When families were asked about the frequency in which they would like various clinical data obtained (e.g. height and weight, PFTs, throat cultures, vital signs, physical exams, labs, and chest x-rays), the responses varied based on the clinical data/test type. In general, most families wanted each of these types of clinical data obtained 1–2 times per year (Figure 2).

### Provider Survey

Of the 25 CF providers who received the provider survey, 24 providers responded (96% response rate). The respondents included: pulmonologists (*n* = 6), gastroenterologists (*n* = 2), pharmacists (*n* = 2), social workers (*n* = 3), respiratory therapists (*n* = 5), physical therapists (*n* = 1), dieticians (*n* = 3), nurses (*n* = 1), and pediatric residents (*n* = 1).

Of the 24 respondents, 21 (88%) were overall satisfied with telemedicine visits and satisfied with the care that they provided. Twenty-two respondents (92%) were satisfied with their experience using Zoom, 18 (75%) were satisfied with their experience using Zoom breakout rooms, and 17 (71%) were satisfied with interpreter experience via Zoom. Despite most providers feeling satisfied with their experience, providers identified various difficulties with telemedicine (Table 2).

**Table 2:** Provider-identified difficulties with telemedicine.

Issues with internet (*n =* 2)
Issues with Zoom (*n =* 2)
Found it difficult to communicate with other providers (*n =* 2)
Difficult to move in and out of different breakout rooms (*n =* 2)
Medical interpreters cannot be called from breakout rooms (*n =* 2)
It is difficult to obtain accurate anthropometric measurements (*n =* 1)
Concerns about the privacy of patients and their families (*n =* 1)

When providers were specifically asked about the number of telemedicine visits they preferred per year, 47% of providers preferred 2 out of 4 recommended annual CF visits be provided virtually. Ninety percent of providers wanted 2 or more visits each year to be telemedicine visits (Figure 1). One respondent (5%) reported wanting all 4 visits to be in-person.

Providers identified the following benefits of visits via telemedicine: decreased cost, decreased travel time, concerns about COVID, concerns about poor air quality from fire smoke, convenience for patients and families (especially those who live far away), and a reduced no show rate. Decreased travel time and concerns about COVID were the two most common reasons that providers wanted to have telemedicine visits. One responder noted that having a separate, additional telemedicine visit to discuss nutrition is sometimes effective because they can spend more time with patients and their families.

The most common reasons providers cited for wanting an in-person visit were: an in-person visit feels more personal, they prefer to do in-person demonstrations/teaching, and it is easier to get labs and tests done on the same day as a visit. Providers also commented that they would like to have in-person visits to perform a physical exam and because it can be hard to tell when patients and families are engaged when having a conversation through a screen.

**Figure 3:**
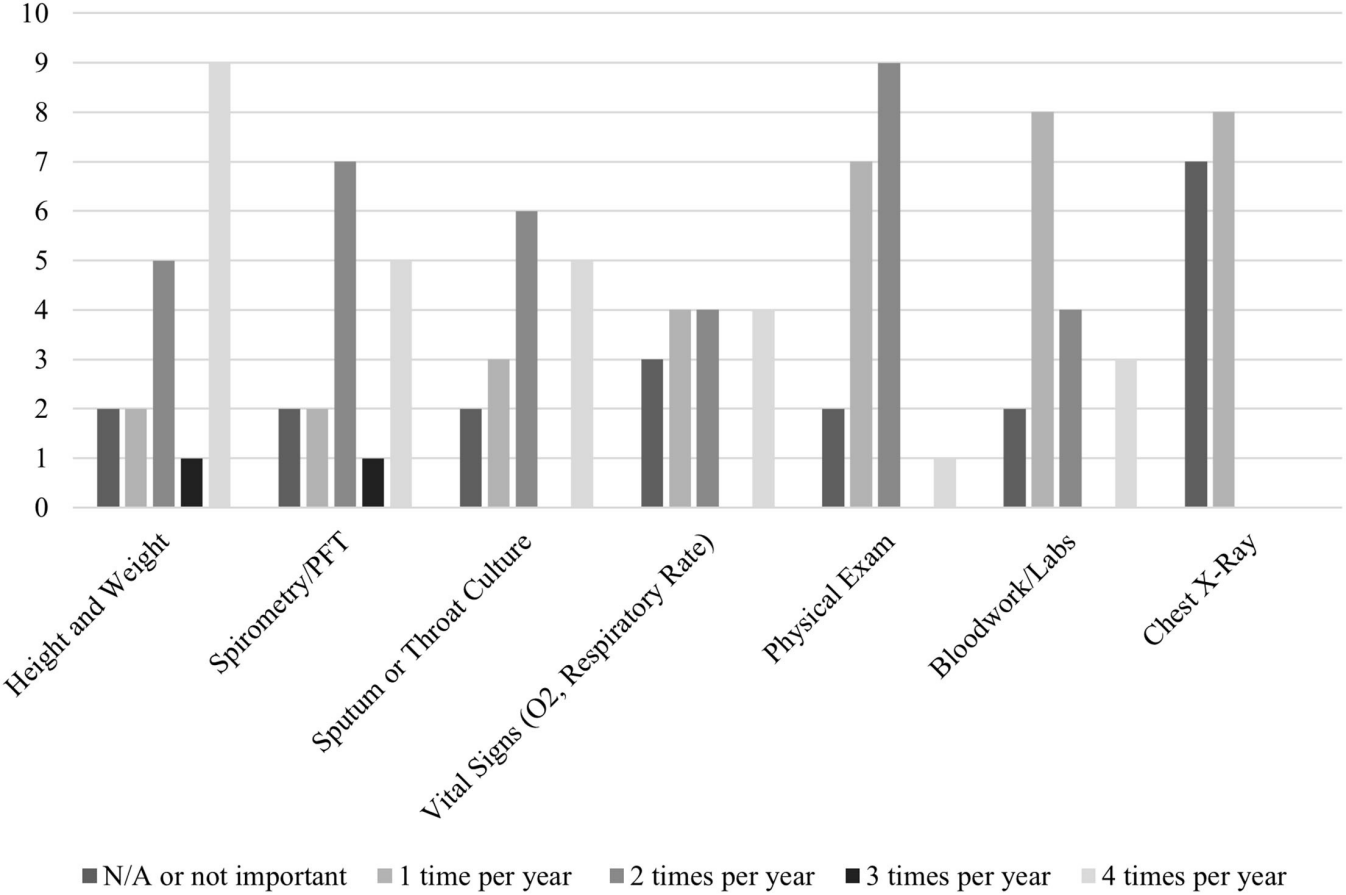
Frequency of obtaining clinical data as desired by providers.

When providers were asked about the frequency in which they would like various clinical data obtained (e.g. height and weight, PFTs, throat cultures, vital signs, physical exams, labs, and chest x-rays), the responses varied based on the clinical data/test type. Overall, most providers wanted each of these types of clinical data to be obtained 1–2 times per year (Figure 3).

## Discussion

Our results demonstrate that 80% of our patients and 88% of our providers were satisfied with their telemedicine visits. Seventy-two percent of families reported wanting to have telemedicine visits in the future, and both patients and providers preferred to have at least 2 of the 4 recommended annual CF visits via telemedicine. These findings are similar to prior studies reporting that the majority of patients and providers want to have future visits via telemedicine (4–6). Interestingly, our data demonstrated a trend of differences between different provider types: respiratory therapists, physical therapists, and social workers tended to prefer more in-person visits; dieticians preferred fewer in-person visits; and physician and nurse providers had varied opinions. Future studies should explore these differences in a larger cohort.

The CF Foundation recommends that all pediatric CF patients have 4 visits per year with a multidisciplinary team of specialists (including pulmonologists, gastroenterologists, pharmacists, social workers, respiratory therapists, physical therapists, dieticians, and nurses) in order to properly care for patients with this chronic and multisystemic disease (7, 8). Because of the number of providers involved in each visit, our visits prior to the pandemic typically lasted over 2 h and sometimes had a significant amount of down-time. With telemedicine, there was less down-time between providers and less time spent rooming patients. During telemedicine visits, families are often more comfortable because they are at home and they do not need to obtain childcare for their other children. Additionally, patients can drift in and out of the visit easily, which allows providers to visually examine patients and ask them questions directly while also minimizing the child's boredom during lengthy visits. This added benefit of telemedicine visits also allows providers to have more focused conversations with the patient's primary caregivers.

The UCSF Pediatric CF Program serves a wide catchment area that spans from the Oregon border to Central California. Our patient population is also diverse: 28% of our patients are non-white and/or Hispanic and approximately 8% of our families speak only Spanish. We pride ourselves on caring for this underserved population and want to ensure that families that live farther away and/or only speak Spanish receive the best quality of care. Some families have previously noted that it is difficult to attend in-person clinic visits due to travel costs and concerns about missing work or school. In our study, we found that most families are satisfied with telemedicine visits and would like to continue using telemedicine after the pandemic. With telemedicine, we can increase access to care for families who live far away or have difficulty coming to in-person visits for various reasons.

In the era of new born screening (NBS) and cystic fibrosis transmembrane conductance regulator (CFTR) modulators, we have seen that our patients are healthier. Even prior to the pandemic, some families were starting to push back on the need for 4 in-person visits each year when they felt their child was healthy. While more research is needed to determine the optimal clinic regimen for the future, and whether family and provider satisfaction changes over time, as the pandemic improves, our data suggests that adding telemedicine as an additional care delivery modality, can improve clinic attendance, and still allow patients and care centers to meet the Cystic Fibrosis Foundation care guidelines.

As we move forward, we must ensure that there is proper support in place to ensure that telemedicine visits go smoothly and that our CF care team members continue to provide high-quality patient care. Some of the logistical components include: providing families with scales and tape measures so they can report accurate height and weight measurements, providing home pulse oximeter devices to measure heart rate and oxygen saturation, home spirometers to remotely monitor FEV1, timely communication with families to ensure they have the appropriate Zoom ID and are able to connect to the internet, and adequate support from medical interpreters. Anecdotally, our low socioeconomic and non-English-speaking families had more difficulty connecting to the Zoom platform, which accounted for the 4 patients who had audio-only telemedicine visits. This lack of video-capability prevented the providers from visually examining the patient during the telemedicine visit. To prevent inadvertently increasing health disparities, we will need to assess each family's access to internet and comfort using Zoom (and other telemedicine technology) before replacing future in-person visits with telemedicine visits.

Some other challenges associated with telemedicine visits include difficulty obtaining lung function testing, throat cultures, and laboratory testing (especially with frequent liver function lab monitoring in the era of CFTR modulator therapy) and providing mental health screening. With access to more reliable portable spirometry devices at home, we may be able to further decrease the number of in-person appointments and decrease travel costs/time for our families. Mental health screening is also difficult to perform via Zoom; it can feel impersonal and there is no guarantee of privacy, as other family members may be able to overhear the conversation. However, we were able to successfully complete mental health screening via video visits and families were able and willing to schedule a separate in-person visits for lung function testing, labs, and respiratory cultures.

One limitation of this study is our small sample size (only 46 families and 24 providers completed our surveys), as this study only involved one CF center. Unfortunately, this made it challenging to draw conclusions from subgroup analyses, including differences in opinions between patients and different provider types. Our study was also limited by our low response rate (only 64% of families responded to the survey), which could have potentially biased our results. Since our survey data was de-identified, we were not able to determine specific characteristics associated with responders vs. non-responders. Communication with families was another limitation; not all families speak English and/or respond reliably to emails. Because of this, our non-English-speaking families completed the survey verbally; the surveys were administered by two research assistants with the help of interpreters. Another limitation was that our patient survey did not include a question regarding patient/family demographics (e.g. ethnicity/race, preferred language, etc.) so we were not able to examine the difference in experience between white and non-white patients/families. Our patient population also includes patients from lower socioeconomic status (many of our patients are on Medicaid), so this data may not be generalizable to other populations. Also, since Zoom was used in this study, our results may not be generalizable to other centers that use other telemedicine platforms.

In conclusion, our study has shown that most families and providers were satisfied with telemedicine and would like to continue using telemedicine in the future. There are several benefits to telemedicine including convenience, decreased travel time and cost, and avoiding exposure to COVID. However, we will need to ensure that we do not inadvertently exacerbate existing health disparities for families that do not speak English and/or do not have the internet capabilities to support telemedicine technology. Future studies are needed to evaluate access and satisfaction of telemedicine technology in patients with racial and ethnic differences, as well as assess satisfaction over time, especially as the pandemic recedes. Further studies are also needed to correlate quality of care with care delivery modalities to develop optimal care delivery guidelines.

## Data Availability Statement

The raw data supporting the conclusions of this article will be made available by the authors, without undue reservation.

## Ethics Statement

Ethical review and approval was not required for the study on human participants in accordance with the local legislation and institutional requirements. Written informed consent for participation was not required for this study in accordance with the national legislation and the institutional requirements.

## Author Contributions

KH was involved in the project's conceptualization, methodology, data collection and analysis, and manuscript writing (original draft and editing). FN was involved in the project's conceptualization, methodology, manuscript writing (editing), and supervision. MC was involved in the project's conceptualization, manuscript writing (editing), and supervision. NL was involved in the project's manuscript writing (editing) and supervision. EG was involved in the project's conceptualization, data collection, manuscript writing (editing), and supervision. All authors contributed to the article and approved the submitted version.

## Conflict of Interest

EG received funding for unrelated studies from Cystic Fibrosis Foundation (CFF-029). NL received funding for unrelated studies from Cystic Fibrosis Foundation (CFF-029), Cystic Fibrosis Foundation Therapeutics (LY18Y0), National Science Foundation (IIP-1622950), HRSA-20-040-172184, and Vertex Pharmaceuticals (VX-7029098). The remaining authors declare that the research was conducted in the absence of any commercial or financial relationships that could be construed as a potential conflict of interest.

## Publisher's Note

All claims expressed in this article are solely those of the authors and do not necessarily represent those of their affiliated organizations, or those of the publisher, the editors and the reviewers. Any product that may be evaluated in this article, or claim that may be made by its manufacturer, is not guaranteed or endorsed by the publisher.
