# Supplementary material for: Patient and Provider Experience With Cystic Fibrosis Telemedicine Clinic
Source: Front Pediatr. 2021 Nov 24;9:784692. doi: 10.3389/fped.2021.784692 (PMC8653948; doi:10.3389/fped.2021.784692)
Supplement: Supplementary file 1 [file Data_Sheet_1.PDF]

CYSTIC FIBROSIS TELEMEDICINE PATIENT SATISFACTION SURVEY

With the COVID-19 pandemic, we have instituted telemedicine to continue to provide care for our patients and families. We would love to hear from you regarding your experience with telehealth and any challenges that you may have faced. Please kindly fill out the survey below so that we can continue to improve our care services. Thank you for partnering with us in your/your child's health.

Please select your/your child's age group:
☐ 0-24 months
☐ 2-5 years
☐ 6-12 years
☐ 13-17 years
☐ 18 years or older

**Satisfaction with Telemedicine Visits**					
Question	Strongly Disagree	Disagree	Neutral	Agree	Strongly Agree
1. I have been overall satisfied with my telemedicine visits.	☐	☐	☐	☐	☐
2. All of my questions and concerns were addressed during my telemedicine visits.	☐	☐	☐	☐	☐
3. I have been satisfied with my experience using Zoom during my telemedicine visits.	☐	☐	☐	☐	☐
4. If you were not satisfied with your experience using Zoom, what was/were the reason(s)? Please select all that apply.	☐ Not applicable – I was satisfied with my experience<br>☐ I had issues with my internet<br>☐ I was not able to connect/had issues with Zoom<br>☐ I do not feel comfortable using telehealth technology<br>☐ Other: _______________________________				

<table>
<tr><th colspan="6">Future Visits</th></tr>
<tr><td>Question</td><td>Strongly Disagree</td><td>Disagree</td><td>Neutral</td><td>Agree</td><td>Strongly Agree</td></tr>
<tr><td>5. I would like to have future visits via telemedicine.</td><td>☐</td><td>☐</td><td>☐</td><td>☐</td><td>☐</td></tr>
<tr><td>6. Of the recommended 4 visits per year, how many visits would you like to have via telemedicine?</td><td colspan="5">☐ 0
☐ 1
☐ 2
☐ 3
☐ 4</td></tr>
<tr><td>7. What is/are the reason(s) you would like to have visits via telemedicine? Please select all that apply.</td><td colspan="5">☐ Decreased cost – do not have to pay for gas and parking
☐ Decreased travel time
☐ Decrease in the amount of time missing work/school
☐ Do not have to arrange for childcare for other children
☐ Concerns about COVID
☐ Concerns about poor air quality from fire smoke
☐ Other: ___________________________________</td></tr>
<tr><td>8. What is/are the reason(s) you would like to have visits in-person? Please select all that apply.</td><td colspan="5">☐ I want a physical examination
☐ I want to do the pulmonary function tests, labs, throat culture, and/or oral glucose tolerance test on the same day
☐ I feel that an in-person visit is more personal
☐ I experienced technical difficulties
☐ Other: ___________________________________</td></tr>
<tr><td rowspan="8">9. Keeping in mind that there are 4 recommended visits per year, it is important to me that the following are measured/obtained in-person with this frequency.</td><td></td><td>N/A or not important</td><td>1 time per year</td><td>2 times per year</td><td>3 times per year</td></tr>
</table>

	N/A or not important	1 time per year	2 times per year	3 times per year	4 times per year
Height and weight	☐	☐	☐	☐	☐
Spirometry/PFT	☐	☐	☐	☐	☐
Sputum culture or throat swab	☐	☐	☐	☐	☐
Vital signs (O2, respiratory rate)	☐	☐	☐	☐	☐
Physical exam	☐	☐	☐	☐	☐
Bloodwork/labs	☐	☐	☐	☐	☐
Chest x-ray	☐	☐	☐	☐	☐

10. Do you have any comments or suggestions about future in-person and telemedicine visits?	
