# Supplementary material for: Patient and Provider Experience With Cystic Fibrosis Telemedicine Clinic
Source: Front Pediatr. 2021 Nov 24;9:784692. doi: 10.3389/fped.2021.784692 (PMC8653948; doi:10.3389/fped.2021.784692)
Supplement: Supplementary file 2 [file Data_Sheet_2.PDF]

CF Telehealth Provider Satisfaction Survey

What type of provider are you?

- ☐ Pulmonary Physician/Fellow
- ☐ GI Physician/Fellow
- ☐ Pharmacist/Pharmacy Resident
- ☐ Social Worker
- ☐ Pediatric Resident
- ☐ Other: ____________________
- ☐ Respiratory Therapist
- ☐ Physical Therapist
- ☐ Registered Dietician
- ☐ Research Coordinator
- ☐ Nurse

Question	Strongly Disagree	Disagree	Neutral	Agree	Strongly Agree
1. I am overall satisfied with CF clinic telemedicine visits.	☐	☐	☐	☐	☐
2. I am satisfied with the quality of care that I provide during CF clinic telemedicine visits.	☐	☐	☐	☐	☐
3. I am satisfied with my experience using Zoom during CF clinic telemedicine visits.	☐	☐	☐	☐	☐
4. I am satisfied with my experience using Zoom breakout rooms during CF clinic telemedicine visits.	☐	☐	☐	☐	☐
5. I am satisfied with my experience interacting with interpreters via Zoom.	☐	☐	☐	☐	☐
6. If you were not satisfied with your experience using Zoom, what was/were the reason(s)? Please select all that apply.	☐ Not applicable – I was satisfied with my experience<br>☐ I had issues with my internet<br>☐ I had issues using Zoom<br>☐ I find it difficult to communicate with other providers via Zoom<br>☐ Other: _________________________				
7. In the future, how many CF clinic visits would you like to have via telemedicine?	☐ 0<br>☐ 1<br>☐ 2<br>☐ 3<br>☐ 4				
8. What are the reasons why you would like to have visits via telemedicine? Please select all that apply.	☐ Decreased cost – do not have to pay for commute and parking<br>☐ Decreased travel time<br>☐ Concerns about COVID<br>☐ Concerns about poor air quality from fire smoke<br>☐ Other: _________________________				

9. What are the reasons why you would like to have in-person visits? Please select all that apply.	☐ I feel that an in-person visit is more personal<br>☐ I prefer to do in-person demonstrations/teaching<br>☐ I prefer to give families handouts in person (as opposed to via email)<br>☐ It is easier to get labs and tests done on the same day as a visit<br>☐ Other: ___________________________					
10. Keeping in mind that there are 4 recommended visits per year, it is important to me that the following are measured/obtained in-person with this frequency.		N/A or not important	1 time per year	2 times per year	3 times per year	4 times per year
	Height and weight	☐	☐	☐	☐	☐
	Spirometry/PFT	☐	☐	☐	☐	☐
	Sputum culture or throat swab	☐	☐	☐	☐	☐
	Vital signs (O2, respiratory rate)	☐	☐	☐	☐	☐
	Physical exam	☐	☐	☐	☐	☐
	Bloodwork/labs	☐	☐	☐	☐	☐
	Chest x-ray	☐	☐	☐	☐	☐
11. Do you have any comments or suggestions about future in-person or telemedicine visits?						
